# Prediction of Sporulation and Germination by the Spider Mite Pathogenic Fungus *Neozygites floridana* (Neozygitomycetes: Neozygitales: Neozygitaceae) Based on Temperature, Humidity and Time

**DOI:** 10.3390/insects9020069

**Published:** 2018-06-19

**Authors:** Thiago Castro, Rafael de Andrade Moral, Clarice Garcia Borges Demétrio, Italo Delalibera, Ingeborg Klingen

**Affiliations:** 1 Department of Entomology and Acarology; ESALQ-University of São Paulo (USP), Av Padua Dias, 11 P.O. Box 9, 13418-900 Piracicaba, Brazil; tcastro@koppert.com.br; 2 Koppert Biological Systems Brazil, Microbiology Product & Development Department, Rodovia Margarida da Graça Martins km 17,5 s/n (SP 135-Estrada Tupi), P.O. Box 35. 13400-970 Piracicaba SP, Brazil; tcastro@koppert.com.br; 3 Department of Mathematics and Statistics, Maynooth University, Maynooth, W23 HW31 Co. Kildare, Ireland; Rafael.DeAndradeMoral@mu.ie; 4 Department of Exact Sciences, ESALQ-University of São Paulo (USP), Av Padua Dias, 11 P.O. Box 9, 13418-900 Piracicaba, Brazil; clarice.demetrio@usp.br; 5 Norwegian Institute of Bioeconomy Research (NIBIO), Division of Biotechnology and Plant Health, Høgskoleveien 7, N-1431 Ås, Norway

**Keywords:** abiotic factors, microbial control, Tetranychidae, Entomophthorales, phytobiome, sporulation, integrated pest management (IPM), Decision Support System (DSS)

## Abstract

*Neozygites floridana* is a pathogenic fungus and natural enemy of the two-spotted spider mite, *Tetranychus urticae* (Acari: Tetranychidae), which is an important polyphagous plant pest. The aim of this study was to reveal and predict what combination of temperature, relative humidity (RH), and time that enables and promotes primary conidia production and capilliconidia formation in *N. floridana* (Brazilian isolate ESALQ 1420), in both a detached leaf assay mimicking climatic conditions in the leaf boundary layer and in a semi-field experiment. In the detached leaf assay, a significant number of conidia were produced at 90% RH but the highest total number of primary conidia and proportion of capilliconidia was found at 95 and 100% RH at 25 °C. Positive temperature and RH effects were observed and conidia production was highest in the 8 to 12 h interval. The semi-field experiment showed that for a >90% probability of *N. floridana* sporulation, a minimum of 6 h with RH >90% and 10 h with temperatures >21 °C, or 6 h with temperatures >21 °C and 15 h with RH >90% was needed. Our study identified suitable conditions for primary- and capilliconidia production in this Brazilian *N. floridana* isolate. This information provides an important base for building models of a Decision Support System (DSS) where this natural enemy may be used as a tool in Integrated Pest Management (IPM) and a base for developing in vivo production systems of *N. floridana*.

## 1. Introduction

The entomopathogenic fungal genus *Neozygites* belongs to the order Neozygitales in the class Neozygitomycetes in the phylum Entomophthoromycota [1]. Fungi in this genus infect small arthropods such as mealybugs, aphids, thrips, and mites [2,3]. Natural epizootics of *Neozygites floridana* have been documented in important mite pests of several major crops [4,5,6,7,8,9,10,11], including the two-spotted spider mite *Tetranychus uricae* (Acari: Tetranychidae), making the fungus an interesting candidate for conservation biological control [12]. However, the use of *Neozygites* in inundative or inoculative biological control is challenging, as *Neozygites* species are biotrophic (obligate pathogens) and are difficult to produce on artificial media [13]. These fungi have therefore only been produced by growing them on the host in small-scale in vivo cultures, even though it has been suggested that it would be possible to produce *Neozygites* on their host on a larger scale, dry the mummies, and store them at a low temperature for later application in inoculative or inundative biological control [14]. *N. floridana* and its related species develop inside spider mites as hyphal bodies, kill their host, penetrate the cuticle, and produce spores (primary conidia). Primary conidia are actively ejected from mite cadavers, referred to as mummies, and these conidia germinate to form the infective capilliconidia that infect new mites [15,16,17]. Environmental factors are known to regulate sporulation and germination of spores of entomopathogenic fungi within the Entomophthoromycota (e.g., [18]) and more specifically within the *Neozygites* (e.g., [19,20,21,22,23]). The ability of a pathogen to form high numbers of infective propagules is very important for the establishment, transmission, and successful epizootic development of the pathogen in a host mite or insect population [24]. One of the limitations for the utilization of *N. floridana* in biological control has been an incomplete understanding of how environmental factors such as relative humidity (RH), temperature, and light affect this [19]. Further, knowledge about optimal abiotic conditions required for *N. floridana* isolates from a specific region is lacking. In addition, the minimum time-period required at these optimal abiotic conditions for sporulation, germination, and infection of the mite to occur is also unknown.

Sporulation studies on microscope slides have shown that RH values greater than 95% and temperatures between 13 and 25 °C are critical for the reproduction of *Neozygites* spp. [16,23,25,26]. Studies on *N. floridana* pathogenic to *Tetranychus evansi* [23] and *Tetranychus urticae* [27] also suggest that the optimal sporulation temperature might vary substantially with the geographical origin of the isolate and the host species. The RH very close to the leaf (in the leaf-boundary layer), where spider mites are typically located, is dependent on leaf transpiration and is often higher than the ambient RH [28]. Macroclimatic conditions that appear to be too dry for mite and insect pathogenic fungi may therefore still provide sufficient humidity for the fungus to sporulate and establish in a host mite or insect population [29]. So far, no study has mimicked leaf-boundary layer conditions or characterized the minimum period of optimal RH and temperature needed for high levels of *N. floridana* primary conidia and capilliconidia production. In our study, we conducted a detached leaf assay mimicking leaf boundary layer conditions to explore (1) the numbers of *N. floridana* primary conidia and capilliconidia produced at four different RHs and temperatures; and (2) the time (hours (h)) at optimal RH- and temperature conditions required for production of a substantial number of *N. floridana* pirmary conidia and capilliconidia. Further, we conducted a semi-field screen house experiment on Jack bean plants where we investigated the correlation of temperature and RH measured in the screen house with the number of *N. floridana* primary conidia and capilliconidia produced.

## 2. Material and Methods

### 2.1. Tetranychus urticae Stock Culture

*Tetranychus urticae* was collected on Jack beans, *Canavalia ensiformis* (Fabales: Fabaceae), in Piracicaba in São Paulo state, Brazil (22°44′ S, 47°38′ W) in 2009. A *T. urticae* stock culture was established and reared on Jack bean plants in 1 L plastic pots, in an acclimatized room at 25 °C, 60% RH, and 12 h of light. The plants were watered five times per week. Old and weak plants were replaced when required.

### 2.2. Neozygites floridana Isolate

The Brazilian *N. floridana* isolate (ESALQ1420) used in these experiments was collected from *T. urticae* on Jack bean plants in Piracicaba, São Paulo state, Brazil (22°44′ S, 47°38′ W) in 2006. Duarte [30] confirmed species-level identification of the isolate using molecular methods.

### 2.3. Cadaver Production

Jack bean leaf discs (1.5 cm diameter) were placed abaxial side up on moistened cotton inside a Petri dish (5 cm in diameter and 2 cm high). Three non-sporulating but *N. floridana*-killed *T. urticae* cadavers were then placed on top of the leaf disc. The Petri dishes with cadavers on leaf discs were then placed inside a plastic box (22 × 16 × 7 cm) and incubated at 25 ± 1 °C and 90% RH for 24 h, to induce sporulation from the cadavers. The boxes were covered with aluminum foil to ensure dark conditions for sporulation. After 24 h, a compound microscope (80×) was used to screen cadavers for sporulation, and only leaf discs with cadavers exhibiting good primary conidia and capilliconidia production were used further. Thirty uninfected adult *T. urticae* females were then transferred to each of the Petri dishes containing a leaf disk and three sporulating cadavers. The Petri dishes were then held at the conditions described above for 24 h to allow *N. floridana* infection of the life *T. urticae*. Petri dishes containing the 30 *N. floridana* inoculated *T. urticae* on leaf discs were then kept at ambient laboratory conditions (21–28 °C, 20–35% RH and 24 h of light) until the *T. urticae* were killed by *N. floridana* (8–9 days). During this period, the lid of the Petri dish was removed to maintain low RH so that *T. urticae* killed by *N. floridana* would not sporulate and rather develop into dry non-sporulating mummies. The cotton in the Petri dish was moistened as required to prevent the leaf disk from wilting. Dry, non-sporulating cadavers were collected from the leaf discs and stored at −20 °C on dry cotton placed on top of blue silica gel (Dinâmica Ltda São Paulo-Brazil) in closed plastic vials (20 mL) for 10–15 days until start of experiment.

### 2.4. Experimental Setup

#### 2.4.1. Detached Leaf Assay: Temperature and RH Combinations

**Figure 1:**
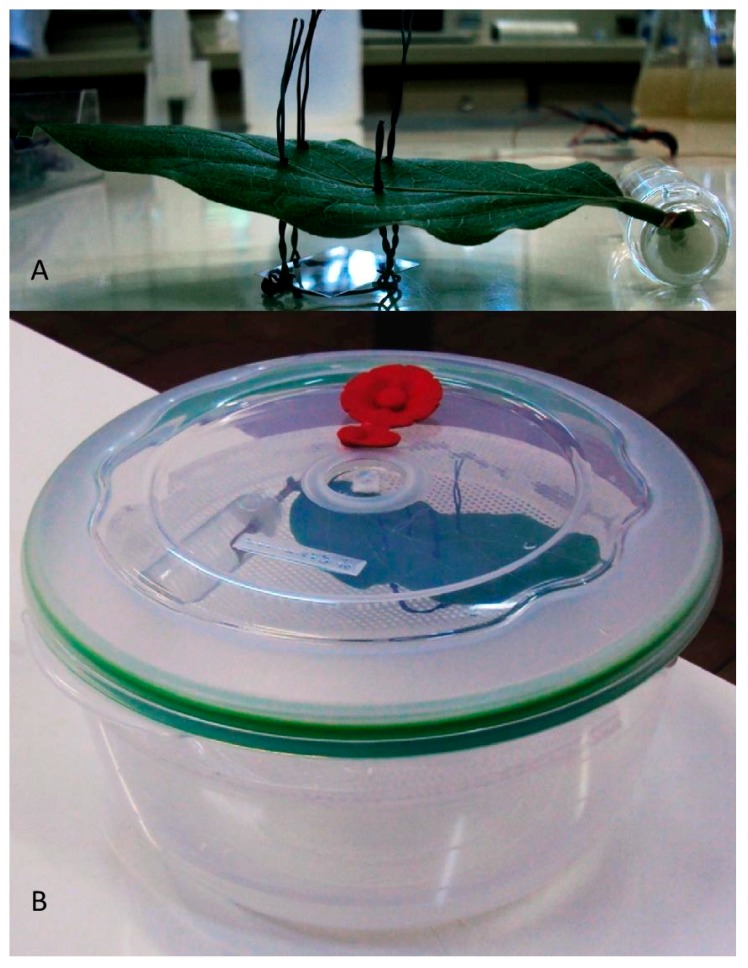
Detached Jack bean leaf assay setup (**A**) The experimental unit: Detached leaf placed above a photo-etched cover slip (18 × 18 mm) with alphanumeric coded squares for collection and counting of conidia discharged from sporulating *Neozygites floridana* killed *Tetranychus urticae* cadavers placed on the underside of the leaf (**B**) The experimental unit placed inside a plastic chamber (22.9 × 21.3 × 12 cm) providing controlled microclimatic conditions.

To determine the optimal microclimatic temperature and RH for *N. floridana* primary conidia and capilliconidia production in a mimicked leaf-boundary layer situation, a detached Jack bean leaf assay was conducted inside a plastic chamber (22.9 × 21.3 × 12 cm—Sanremo^®^, Flower line). The chambers contained 350 mL of a sulfuric acid solution at appropriate concentrations to obtain the targeted RHs as described by Solomon [31] and were placed in climatic chambers to obtain the desired temperatures (Figure 1). Five RH levels (80, 85, 90, 95 and 100%) in combination with four temperature levels (13, 17, 21 and 25 °C) were tested. Temperature levels were chosen based on the average yearly temperatures observed between 18:00 and 06:00 in Piracicaba, São Paulo, Brazil (http://www.ler.esalq.usp.br/posto.html). A digital thermal hygrometer (Instrutherm—HT-500) was used inside the plastic chamber to confirm RH and temperature were accurate. Each experimental unit consisted of a Jack bean leaf with its petiole inserted into a 15 mL glass tube filled with distilled water and sealed with paraffin, to prevent the petiole from moving and evaporation of the water (Figure 1). Two *N. floridana* killed and mummified *T. urticae* females were placed on the abaxial side of each leaf. A photo-etched cover slip (18 × 18 mm) with alphanumeric coded squares (Electron Microscopy Sciences, Hatfield, PA, USA) was placed 1 cm below the leaf and cadavers in order to collect conidia. The experimental unit was kept in darkness inside the chamber for 12 h. Numbers of primary conidia and capilliconidia were counted on each photo-etched coverslip surface using a phase contrast microscope (×400). Primary conidia that were in the process of forming or had already formed a capillary tube were defined as capilliconidia, all others were considered primary conidia. Five replicates of each RH × temperature combination were performed yielding a total of 10 mummies per treatment and 160 mummies for the entire experiment.

#### 2.4.2. Detached Leaf Assay: Minimum Period at Optimal Conditions for Sporulation

To determine the minimum period (hours) needed at optimal RH and temperature conditions for substantial production of *N. floridana* primary conidia and capilliconidia, we used the same assay setup described above. Three humidity levels (90, 95, and 100% RH) were used in combination with two temperature levels (13 °C and 25 °C). Cadavers were exposed to these conditions for five different periods of time (4, 8, 12, 24, and 36 h). Four replicates of each RH × temperature × duration combination yielded a total of 16 cadavers per combination and a total of 480 cadavers used in the experiment.

#### 2.4.3. Semi-Field Screen House Experiment

A screen house experiment was conducted to investigate the correlation between temperature and RH under semi-field conditions and the number of *N. floridana* primary conidia and capilliconidia produced. Five *N. floridana* killed and mummified *T. urticae* female mites were placed onto each of two leaves of a three-week old Jack bean plant once a week. Cadavers were placed on the underside of the leaf, near the central leaf vein, and at a distance of three cm from one another. To enable collection of conidia, leaves were taped to a plastic support with the abaxial side facing up. Plants were grown in 1L plastic pots and the experiment was conducted from August 2010 to November 2011 in a screen house located at ESALQ-USP in Piracicaba, São Paulo, Brazil (22°44′ S, 47°38′ W). Leaves with mummies were destructively sampled from the plant 24 h after cadaver introduction. Primary conidia and capilliconidia produced was assessed by cutting out a leaf disc 2 cm in diameter around the cadaver. The cadaver was then removed and the leaf disc was placed on a glass slide under a microscope (×100) the primary conidia and capilliconidia were counted using the same criteria described above. The light from the microscope made the leaf transparent but conidia visible. To facilitate large scale counting of conidia, we used a categorical scale as described by Wekesa [23] where high >500 conidia, low >1 and <500 conidia, and zero=no conidia. RH and temperature data were recorded using a data logger (HT-500, Instrutherm-São Paulo, Brazil) located close to a Jack bean leaf inside the screenhouse.

### 2.5. Statistical Analysis

The statistical analyses were based on the theory of generalized linear models and extensions [32]. All analyses were carried out in R, using the “stats” package [33]. Half-normal plots with simulation envelopes were used to assess goodness-of-fit of the fitted models, using the package “hnp” [34].

#### 2.5.1. Detached Leaf Assays: Temperature, RH, and Duration

##### Number of Primary Conidia and Capilliconidia, Total Number of Conidia, and Proportion of Capilliconidia

Quasi-Poisson generalized linear models were fitted to the count data, with different intercepts and slopes over temperature per RH level. Quasi-binomial generalized linear models were fitted to the proportion of germinated conidia out of total number of produced conidia (primary conidia + capilliconidia), with different intercepts and slopes over temperature per RH level. Nested models were also fitted to the data and F-tests were performed to assess the significance of the effects.

#### 2.5.2. Semi-Field Screen House Experiment

A quasi-binomial generalized linear model was fitted to the proportion of cadavers that sporulated (scored as “low” or “high” sporulation based on the categorical scale described above) including a response surface for the time spent (in hours) at RH higher than 90% and temperature higher than 21°C.

##### Analyses for Experiment Considering Duration

For the total number of conidia (primary conidia + capilliconidia), as well as number of primary conidia and capilliconidia, quasi-Poisson generalized linear models were fitted with different linear predictors per each temperature × RH combination, with a segmented regression over time. The breakpoint of the segmented regression was estimated by maximizing the profile-likelihood of a Poisson model fitted using the maximal linear predictor described above. Nested models were also fitted to the data and F-tests were performed to assess the significance of the effects.

##### Goodness-of-Fit Assessment and Software

Half-normal plots with simulation envelopes were used to assess goodness-of-fit of the fitted models, using package “hnp” [34]. All analyses were carried out in R, using the “stats” package [33].

## 3. Results

### 3.1. Detached Leaf Assay: Temperature and RH Combinations

**Figure 2:**
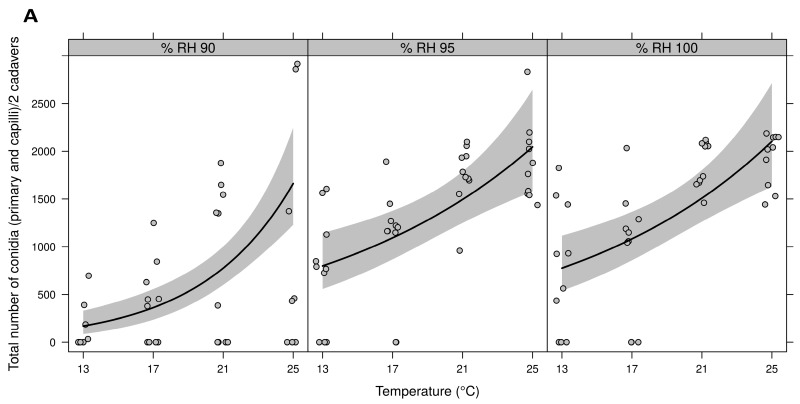

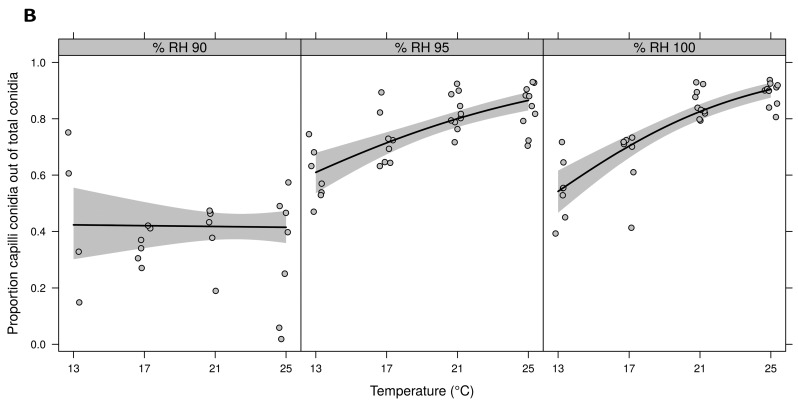
Effect of relative humidity (RH) (80, 85, 90, 95, 100%) and temperature (13, 17, 21, and 25 °C) on (**A**) Mean total number of conidia (primary conidia and capilliconidia)/cadaver for a Brazilian *Neozygites floridana* isolate (ESALQ 1420) (**B**) Proportion of *Neozygites floridana* capilliconidia out of the total number of conidia. RH 80% and 85% not shown, as no sporulation was detected.

Figure 2A demonstrates that the highest number of conidia (primary conidia and capilliconidia) was produced at 95% and 100% RH at 25 °C. Also, at 13, 17, and 21 °C the number of primary conidia and capilliconidia produced was significantly (F_2,114_ = 4.74, *p* = 0.0106) higher at ≥95% RH than ≤90% RH. Further, a significant (F_1,118_ = 59.21, *p* < 0.0001) positive temperature effect on total conidia production was seen for all temperatures at ≥90% RH (Figure 2A). Figure 2B suggests that the highest proportion of capilliconidia was produced at ≥95% RH at 25 °C. The same trend occurred at lower temperatures (13, 17, and 21 °C), with ≥95% RH resulting in a significantly (F_2,86_ = 12.83, *p* < 0.0001) higher proportion of capilliconidia produced compared to lower RHs. Further, a significant (F_1,90_ = 31.36, *p* < 0.0001) positive temperature effect on proportion of capilliconidia produced was seen for all temperatures at ≥95% RH (Figure 2B). Capilliconidia production was influenced more by RH than primary conidia production, and at 90% RH, an increase in capilliconidia production with increased temperature was not observed even though it was observed for primary conidia (Figure 2A,B). No sporulation occurred at any temperature when RH was ≤85% (Figure 2A).

### 3.2. Detached Leaf Assay: Minimum Period for Optimal RH and Temperature

**Figure 3:**
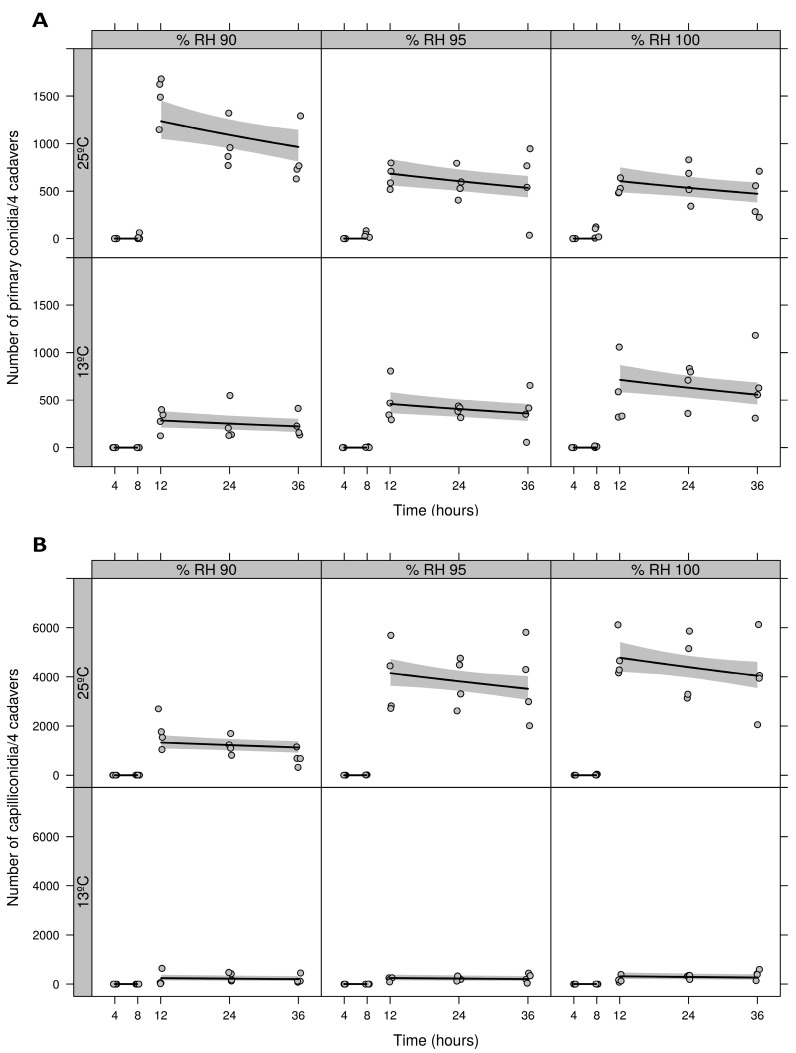
Effect of time (4, 8, 12, 24 or 36 h) on primary conidia production (**A**) and capilliconidia formation (**B**) by a Brazilian *Neozygites floridana* isolate (ESALQ 1420) at three different RHs (90, 95, 100%) and two temperatures (13, 25 °C).

Almost all primary conidia were discharged between 8 and 12 h (estimated breakpoint for the segmented regressions) for all combinations of environmental factors tested (Figure 3A). For primary conidia there was a significant interaction between RH and temperature (F_2,112_ = 33.15, *p* < 0.0001), showing that all curves were different, except for the temperature 25 °C, where 95% RH and 100% RH are statistically equal (F_1,113_ = 0.79, *p* = 0.3751) (Figure 3A). Capilliconidia formation was also observed only between 8 and 12 h for all combinations of environmental factors tested (Figure 3B). For capilliconidia there was a significant interaction between RH and temperature (F_2,112_ = 6.06, *p* = 0.0032), except for at 95% RH and 100% RH which independent of the temperature, were equal (F_2,112_ = 2.10, *p* = 0.1267) (Figure 3B). After 12, 24, and 36 h at 25 °C, a higher number of primary conidia was observed at 90% RH compared to 95% and 100% RH (Figure 3A) while for capilliconidia, a lower number was observed at 90% RH compared to 95% and 100% RH (Figure 2B). At 13 °C, formation of capilliconidia from primary conidia was close to zero (Figure 3B). Further, no sporulation was observed from mummified mites during the first 4 h or after 12 h for any of the environmental conditions tested (Figure 3A).

### 3.3. Semi-Field Screen House Experiment

**Figure 4:**
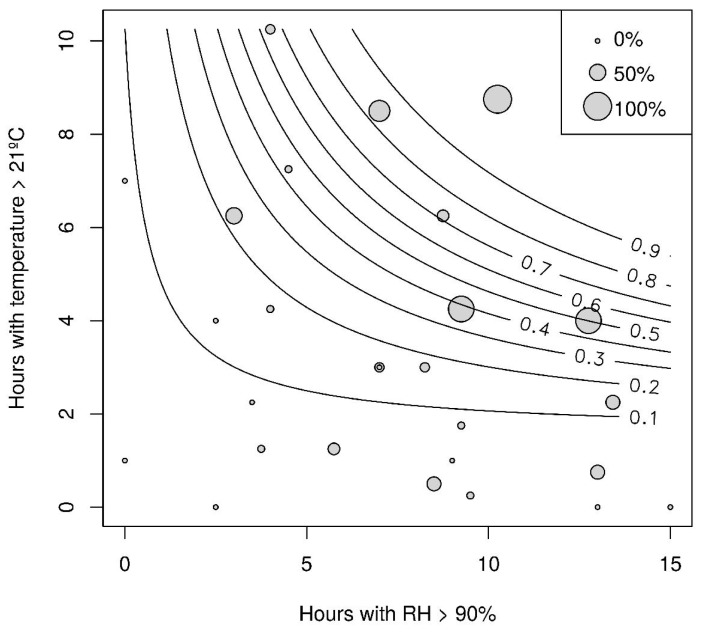
Combined effect of temperature and RH on sporulation of a Brazilian *Neozygites floridana* isolate (ESALQ 1420) from *Tetranychus urticae* cadavers on Jack beans in a semi-field screen house study in Piracicaba, São Paulo, Brazil, from August 2010 to November 2011. Point size is scaled by the percentage of cadavers sporulating and the lines represent the probability of sporulation from 0.1 to 0.9 (10% to 90%).

The screen house semi-field experiment showed a clear positive correlation between number of hours with RH >90% and number of hours with temperatures >21 °C and when a combination of both these factors were present, a higher sporulation of the Brazilian *N. floridana* isolate tested occurred. For >90% probability of *N. floridana* sporulation, a minimum of 6 h with RH >90% (F_1,25_ = 12.97, *p* = 0.0014) and 10 h with temperatures >21 °C (F_1,26_ = 9.36, *p* = 0.0054), or a minimum of 6 h with temperatures >21 °C and 15 h with RH >90% (Interaction: F_1,24_ = 9.17, *p* = 0.0058), was needed (Figure 4).

## 4. Discussion

Under the leaf-boundary layer climatic conditions mimicked in our detached leaf assay we found that the Brazilian *N. floridana* isolate sporulated at 90% RH and that, at 25 °C, no difference in the number of primary conidia and capilliconidia produced was observed between 95% and 100% RH. Previous studies aiming to determine the temperature and RH requirement for sporulation of *Neozygites* spp. were carried out on microscope slides and have shown that RH values greater than 95% (100% being the best RH) and temperatures between 13 and 27 °C are critical for fungal reproduction [16,23,25,26,35].

The optimal temperature for conidia production found here concurs with the optimal temperature 25 °C that Wekesa [23] found for two Brazilian *N. floridana* isolates on *T. evansi* (ESALQ 1419 and ESALQ 1421) tested at 13, 17, 21, 25, 29, and 33 °C. It is, however, higher compared to the optimal temperature of 18 °C found for a Norwegian *N. floridana* isolate on *T. urticae* (NCRI 271/04) tested at 13, 18, and 23 °C [27]. Neither Wekesa [23] nor Klingen and Nilsen [27] tested the effect of different RH levels. Smitley et al. [35] report that, when testing numbers of primary conidia produced by a *N. floridana* isolate (host *T. urticae*) from Chowan, North Carolina, USA at 4, 10, 16, 21, 27, 29, and 32 °C and 75%, 85%, 98%, and 100% RH, the temperature optimum was 21 °C at 100% RH. A significant number of primary conidia was also produced at 98% RH but not at 75 or 85% RH. Smitley et al. [35] also report that temperature and RH requirements for the formation of capilliconidia were similar for the production of primary conidia. Our studies suggest, however, that capilliconidia production was more influenced by RH than primary conidia production. Therefore, further detailed study is needed to fully understand the effect of abiotic factors and mechanisms by which they impact primary conidia and capilliconidia production in this fungus. Brown and Hasibuan [25] also report that the highest number of *N. floridana* conidia produced (not distinguishing between conidial type) on *T. urticae* (fungus and host originating from North Carolina) were at 100% RH when compared to 90% and 80% RH. In addition, studies conducted with *Neozygites tanajoae* (earlier named *N. floridana*) on *Mononychellus tanajoa* (e.g., [16,36,37]) suggest that RH levels above 96% were needed before primary conidia production occurred.

When comparing different studies with *N. floridana* we suggest that the optimal temperature varies depending on the geographic, and hence climatic, origin of the isolate tested. It ranges from 18 °C for isolate from a northern location like Ås, Norway (59°39″ N, 10°46′ W) through 21 °C for isolate from a temperate location like Chowan, North Carolina, USA (38°07″ N, 76°35′ W) to 27 °C from a tropical location like Piracicaba, São Paulo, Brazil (22°44′ S, 47°38′ W) in this study. The average temperature during the growth season for these three locations is in the same range as the optimum temperature for the fungus. We therefore suggest that to be able to build good descriptive models for this pest natural enemy system it is imperative to include knowledge about the temperature in the region where the isolate come from. The optimal RH levels are more similar for all geographical origins, however, and close to 100%. In our detached leaf assay mimicking leaf boundary climatic conditions, sporulation was found at ambient RH conditions of 90% which is lower than what has been found in laboratory studies measuring sporulation on microscopic slides. This may suggest that the humidity in the microclimate close to leaf surface (in the leaf boundary layer) in our study was higher and that this might have contributed to the primary conidia production. Tomato greenhouse studies with fungi in the Hypocreales support that a higher humidity is found in the leaf boundary layer and that this benefits microbial control of small arthropod pests, such as *T. urticae*, living in the leaf boundary layer [28,29,38].

In the laboratory time series assay, almost all primary conidia were discharged and most capilliconidia formed between 8 and 12 h under the environmental factors tested (RH and temperature). Our screen house semi-field experiment with the same Brazilian *N. floridana* isolate confirms the findings from our assays mimicking leaf-boundary climatic conditions. This provides preliminary evidence that our calculated optima can be applied in natural field settings, but further experimental field data is required to confirm this. Further, our screen house semi-field experiment that indicates the duration of the period with optimal RH level and temperature is also relevant to conidia production. A clear positive correlation was observed between a combination of number of hours (minimum 6 h) with RH >90% and number of hours (minimum 10 h) with temperatures >21 °C for high sporulation. As such, the duration of periods within the temperature and RH optimal ranges should be included when building a descriptive model for this pest natural enemy system. 

This study identifies both temperature and RH optima for conidia production in *N. floridana*, as well as suggesting the minimum duration of these conditions needed to result in significant sporulation. It is important, however, to note the limitations for the implications of the results from one isolate to another isolate, as the optimal temperature for conidiation in *N. floridana* varies and seems to be linked to the geographical and climatic origin of the isolate. This needs to be taken into consideration when carefully making descriptive models for the potential epidemic development for the fungus in a specific host pathogen system in a specific climatic region (e.g., tropical or temperate) or production system (e.g., field or greenhouse). Detailed characterization of the abiotic factors affecting reproduction of insect pathogens provide an important base for building descriptive models with potential to be further developed, validated, and used in a Decision Support System (DSS) for the use of this natural enemy as a tool in Integrated Pest Management (IPM). The data presented here also provides a basis that could be used for establishing an in vivo biocontrol agent production system for *N. floridana*.

## 5. Conclusions

Significant number of conidia were produced at 90% RH but the highest total number of primary conidia and proportion of capilliconidia was found at 95 and 100% RH at 25 °C. Most conidia were produced between 8 and 12 h. Based on the semi-field experiment it is predicted that there was 90% probability for *N. floridana* sporulation to occur, with a minimum of 6 h with RH >90% and 10 h with temperatures >21 °C; or 6 h with temperatures >21 °C and 15 h with RH >90% needed. This information provides an important base for building models of a pest natural enemy Decision Support System (DSS) and for developing in vivo production systems for *N. floridana*.
